# Spinel-Type Materials Used for Gas Sensing: A Review

**DOI:** 10.3390/s20185413

**Published:** 2020-09-21

**Authors:** Yudong Li, Zhenyu Yuan, Fanli Meng

**Affiliations:** College of Information Science and Engineering, Northeastern University, Shenyang 110819, China; 1970652@stu.neu.edu.cn

**Keywords:** spinel structure, nanomaterials, gas sensing, gas mechanism, doping

## Abstract

Demands for the detection of harmful gas in daily life have arisen for a period and a gas nano-sensor acting as a kind of instrument that can directly detect gas has been of wide concern. The spinel-type nanomaterial is suitable for the research of gas sensors because of its unique structure. However, the existing instability, higher detection limit, and operating temperature of the spinel materials limit the extension of the spinel material sensor. This paper reviews the research progress of spinel materials in gas sensor technology in recent years and lists the common morphological structures and material sensitization methods in combination with previous works.

## 1. Introduction

### 1.1. Gas Nano-Sensor

It is easy to encounter some flammable and toxic gases in daily life, some of which can cause air pollution and affect human health [1]. To detect the specific gas, chemical gas sensors have drawn the attention of scientists for a long period. Up to now, the gas sensing devices based on metal oxide semiconductors (MOS) have been wildly used owing to several advantages, such as their low cost, lower service restrictions, and long stability [2]. Properties of a gas sensor are evaluated by parameters such as response, operating temperature, repeatability, response/recovery time, detection limit, and selectivity. These important indexes are usually related to the sensitive material of the sensor, so approaches to more sensitive materials have been studied for a long time.

Nanomaterials refer to materials with at least one dimension of nanometer size (1–100 nm) in the three-dimensional space. According to the dimension of the nanoscale, nanomaterials can be divided into zero-dimensional structure, one-dimensional structure, and two-dimensional structure. It should be noted that a three-dimensional structure refers to a structure formed by low-dimensional materials, which still belongs to nanomaterials. In continuous in-depth research, nanomaterials are considered to be more suitable for gas sensing than traditional materials. Because nanomaterials will produce unique characteristics because of nanometer effects such as small size effects, surface and interface effects, and macroscopic quantum tunneling effects [3]. Therefore, the materials involved in this article are all in the nanometer size range.

### 1.2. Spinel-Type Materials

**Figure 1:**
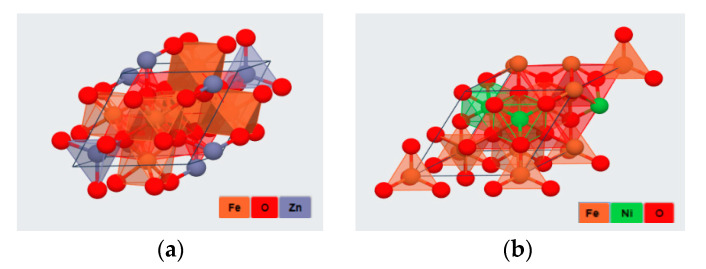
(**a**) Normal structure: ZnFe_2_O_4_; (**b**) Inverse structure: NiFe_2_O_4_. The atoms are represented by spheres: Fe (orange), O (red), Zn (purple) and Ni (green).

Spinel structure can be divided into many types according to the non-metal elements, including oxides, sulfides, fluorides, etc. [4] and the most common oxides are illustrated as examples herein. The common formula for normal and inverse spinel structures are A^tetra^(B_2_)^octa^O_4_ and B^tetra^(AB)^octa^O_4_ respectively [5]. In the structure of a normal spinel material, A is a divalent metal ion, which generally refers to the elements like Zn, Cd, Cu, Ni, Mg, and Co placed at the tetrahedral site. Meanwhile, the component B (usually means the element Fe, Cr, Ga, Co, and Al [6]) is the trivalent ion placed at the octahedral site. In Figure 1a, oxygen ions arrange in cubic close packing, while the divalent cations fill in one-eighth tetrahedral void and trivalent cations fill in a half octahedral void.

Many investigators have been exploring the potential of spinel materials in gas and catalysis fields because of its specific 3D porous structure for some time. As a semiconductor, spinel materials have been studied as an electronic material for over fifty years [6]. Up to now, spinel structures have been widely used in gas storage or separation [7,8] and battery catalysis [9,10], but remain less popular in the gas sensing field. Most recently, the reviews mainly follow closely to the gas chemo catalysis and gas absorption [11], several recent articles have reviewed the research progress on spinel structures chemical using without special attention on gas sensing. The articles which concentrate on the spinel-type material synthetic method and improvement of gas sensing only have not been reviewed in the literature before. At present, some existing studies indicate that spinel materials can also be widely used in gas detection and its special structure has aroused people’s interest in the gas sensing field. Therefore, we attempt to force on the gas sensing aspect in this article. In this review, we make efforts to provide an overview of the spinel materials for structure, geometry, and modification in the gas sensing field.

## 2. Sensing Mechanism

### 2.1. N-Type Material

In this section, a typical n-type semiconductor ZnFe_2_O_4_ will be analyzed to clarify the agreed-upon working mechanism of a chemical gas sensor. According to habit, the change of the resistance, usually written as *R*_a_/*R_g_* (*R_a_*: the resistance in the air; *R_g_*: the resistance of the sensor after exposing to the aimed gas), is the response which is the main performance of a gas sensor.

When the ZnFe_2_O_4_ based sensor is exposed to the air, the oxygen molecules in the air will chemisorb onto the metal oxide particle capturing the electrons and transform into O_2_^−^, O^−^, or O^2−^, which specifically depend on the temperature shown in Equations (1)–(4). The electron transfers that occur in Equations (2)–(4) cause the formation of the depletion layer on the material’s surface, making the conductive channel narrow with the increase of R_a_ [12].

(1) $$O_{2(gas)}↔O_{2(ads)}\;$$

(2) $$O_{2(\mathrm{ads})}+e^{-}↔{{O_{2}}^{-}}_{\left(\mathrm{ads}\right)}\;\;\;\;\;\;\;\;\;\;\;\;\;\;\;\;\;\;\;T<147\;{}^{\circ}C$$

(3) $${{O_{2}}^{-}}_{\left(\mathrm{ads}\right)}\;+e^{-}↔2{O^{-}}_{\left(\mathrm{ads}\right)}\;\;\;\;\;\;\;\;\;\;\;\;\;\;147{}^{\circ}C<\;\;T<397\;{}^{\circ}C$$

(4) $${O^{-}}_{\left(\mathrm{ads}\right)}\;+e^{-}↔{O^{2-}}_{\left(\mathrm{ads}\right)}\;\;\;\;\;\;\;\;\;\;\;\;\;\;\;\;\;\;\;T>397\;{}^{\circ}C$$

Considering the particularity of spinel-type structural materials, it also has a different principle from ordinary single metal oxides. During the sensing process, the chemisorbed oxide particle can capture electrons, changing Fe^2+^ to Fe^3+^, which is shown in Equation (5). On account of the dissociation effect of oxygen, the valency distribution and defects in the crystal structure are changed, while a part of the conductive three-dimensional network structure is cut off so that the Ra of the material increases significantly [12].

(5) $${\mathrm{Fe}}^{3+}+e^{-}↔{\mathrm{Fe}}^{2+}$$

When the reduced gas diffuses to the surface of the sensitive material, it will rapidly undergo a redox reaction with the oxygen on the surface. During the reaction, electrons captured by oxygen will return to the material, and the resistance will be obviously reduced. According to the sensing mechanism, the possible reacting processes to reduced gas are as follows Equations (6) and (7) [13], while the detailed reaction will change with the operating temperature. For example, when a spinel material detects ethanol at 147–397 °C, there will be a reaction, as shown in Equation (8). The electrons will be released back to the surface first and combine with the surface vacancy that rapidly decreases the resistance of the sensor as well as reduce Fe^3+^ to Fe^2+^.

(6) $$G_{\left(\mathrm{gas}\right)}↔G_{(\mathrm{ads})\;}$$

(7) $${G_{\left(\mathrm{ads}\right)}\;+\;O}^{-}\to {\mathrm{GO}\;+\;e}^{-}\;\;\;\;\;\;147\;{}^{\circ}C\;<\;T\;<\;397\;{}^{\circ}C$$

(8) $${\mathrm{CH}}_{3}{\mathrm{CH}}_{2}{\mathrm{OH}\;+\;8O}^{-}\to 3{\mathrm{CO}}_{2}{+3H}_{2}{O\;+\;8e}^{-}\;\;\;$$

### 2.2. P-Type Material

The mechanism of p-type material, NiFe_2_O_4_, is easily understood by analogy. It should be noted that the response this time is defined as *R_g_*/*R_a_*. In the reduced reaction, the hole becomes the carrier, which means the change of resistance presents the opposite trend of the n-type material. Due to the suspicion, the transformation in the process might react as follows [14,15] in Equation (9):

(9) $${\mathrm{Ni}}^{2+}{+\;h}^{+}↔{\mathrm{Ni}}^{3+}$$

### 2.3. Mixed-Type Material

So far, there is no systematic research on the mixed type material because of its complexity. The mixed-type material generally includes more than two kinds of cations such as Ni-Zn ferrites (Chemical formula: Ni_1-x_Zn_x_Fe_2_O_4_) and Co-Ni ferries (Co_1-x_Ni_x_Fe_2_O_4_). In the synthesis of nickel-zinc ferrite, zinc ions prefer to occupy tetrahedral positions than iron ions, along with the replacement of the Fe^3+^ ions from tetrahedral to octahedral positions, forming a kind of mixed-typed spinel material with divalent and trivalent ions distributed uniformly in the tetrahedron and octahedral position. Properties of the mixed-type material often depend on the operating temperature. For example, the Ni_0.5_Zn_0.5_Fe_2_O_4_ and Ni_0.3_Zn_0.7_Fe_2_O_4_ will show the gas sensing characteristics of the n-type material when the temperature is lower than 225 °C, while it will show characteristics of the p-type structure material when it is higher than 225 °C [6]. The performance of the mixed type spinel structure is greatly affected by cations composition, synthesis process and test conditions, so there is no unified sensing mechanism has been found. However, the formation of the mixed-type structure has an uncertain effect on the gas sensing properties of the material. In the enhancing chapter, the effect of metal doping will be introduced later.

## 3. Morphology

In the process of improving gas sensor performance, the morphology of sensitive materials is a primary improvement direction. Reasonable specific surface area, porosity, growth direction and grain size are related to the sensitivity and other properties of the gas nano-sensor. Therefore, the controllable morphology of spinel materials is not only its advantages but also the direction that researchers have been working hard on. Using different preparation methods, the researchers obtained several spinel materials with different morphologies. The following sections will review the morphology of the spinel-type materials common in the literature and their advantages of gas sensing brought about by the diverse morphology.

### 3.1. Nanoparticles

The morphology of nanoparticles determines that electrons need to pass through more interfaces during the electron transport process, which will reduce the efficiency noticeably. Further in the synthesis process, zero dimensional nanoparticles more easily form agglomerates, which increases the difficulty of synthesis. Although the particle morphology has a larger specific surface area, it is still not suitable to the improvement of gas sensitivity. In short, the spinel structure of nanoparticles is not the most ideal morphology for gas sensing detection, and relatively fewer documents also illustrate this point.

**Figure 2:**
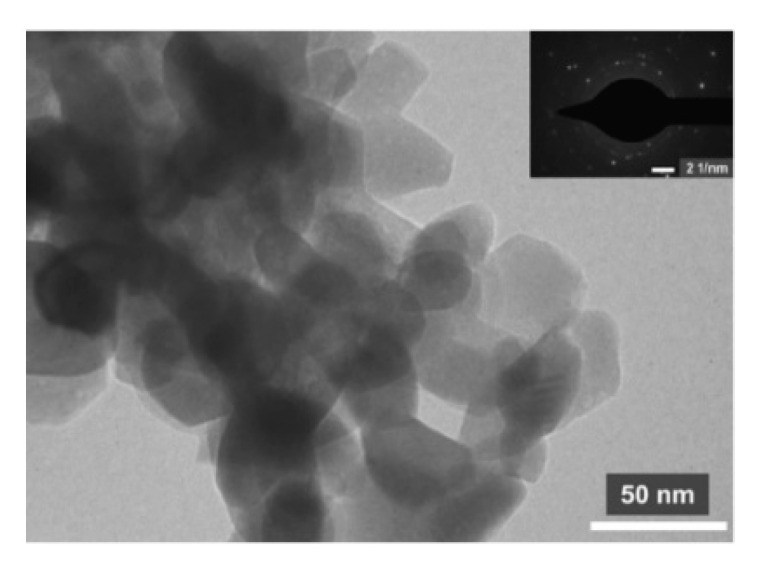
TEM and SAED images of ZnFe_2_O_4_ powder [17].

Patil et al. [16] used the glutamic acid combustion method to synthesize the ZnFe_2_O_4_ powder structure. During the process, by mixing Zn (NO_3_)_2_·6H_2_O, Fe (NO_3_)_3_·9H_2_O and glycine, sparks were generated when heated to the critical temperature and the burned product formed into brown fluffy ZnFe_2_O_4_ powder. Combined with the results of the expected morphology shown in Figure 2, the advantages of the combustion synthesis method, like the convenience, environmental protection, economy, and high efficiency [17], mean it should be followed up to a certain extent in the synthesis of materials.

**Figure 3:**
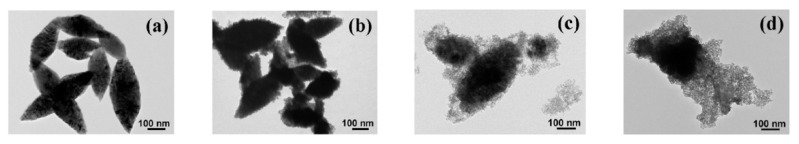
TEM images of the sample synthesized with different mole ratios of ZnO to FeCl_3_: (**a**) 1:0, (**b**) 6:1, (**c**) 3:1 and (**d**) 1:1 [18].

Zhang et al. [18] synthesized ZnFe_2_O_4_ nanoparticles with the hydrothermal method which makes the material present a good dispersion and a better sensing property to acetone. The experimental reaction conditions have a crucial influence on the morphology, and this factor will be discussed in the subsequent enhancing section. By continuously changing the molar ratio of zinc oxide and ferric chloride and the reaction time, the experiment had obtained several morphologies represented in Figure 3. Here, the optimal morphology was synthesized by following conditions: molar ratio 1:2, reaction time over 12 h and the reaction temperature at 180 °C. Further from the gas response curves in the article, the ZnFe_2_O_4_ based gas sensor had a higher response (39.5) than the zinc oxide sensor (4.2) in the same testing condition with lower operating temperature at 200 °C. Therefore, we can reasonably speculate that zinc ferrite material is an ideal alternative material for detecting acetone.

**Figure 4:**
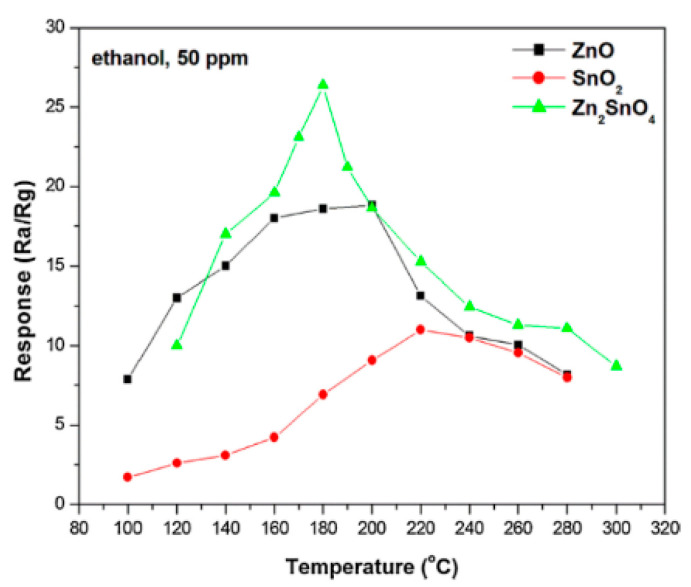
Response of the calcined ZnO, SnO_2_, and Zn_2_SnO_4_-based sensors to 50 ppm ethanol gas at different working temperatures [19].

For more in-depth study of the material advantages, An et al. [19] synthesized zinc oxide, tin oxide and zinc stannate (a typical inverse spinel structure) via co-precipitation methods. Through analyzing the N_2_ adsorption-desorption isotherms and the pore size distribution curve, it can be confirmed that the synthesized material this time had a typical porous structure. In order to further determine the performance of the material, gas sensitivity tests were carried out onto the three materials. The results in Figure 4 showed an obvious gap that zinc stannate reached a response of 39.5 to ethanol gas at a lower working temperature (180 °C), which was comparable to zinc oxide (18.6, 200 °C) and tin oxide (15.3, 240 °C) in terms of performance.

### 3.2. Nanorods

Connected structure like nanowire or nanorod means fewer contact interfaces during electron transmission. This kind of one-dimensional (1D) avoids the recombination of electrons with other ions and enables electrons to be transferred at a higher speed. At the same time, one-dimensional materials are more beneficial to complete the contact with reactants, making nanowire/nanorod more suitable for gas sensors.

Ponmudi et al. [20] used sputtering technique to synthesize the nano-grass morphology of copper aluminate (CuAl_2_O_4_, which has been less studied) for the gas sensing. Experimental data showed that the material could produce 99.77 response to 100 ppm ammonia gas, and there was still a certain response to concentration below 10ppm. The good selectivity and high response indicate that the material has certain research prospects and is worthy of further research. Mintcheva et al. [21] also made certain contributions to the detection of ammonia. The researchers used the laser ablation in liquid (LAL) method to synthesize hybrid oxide nanoparticles of zinc and tin and conducted simple tests. The results showed that the synthesized material could produce a 92% response to 250 ppm ammonia at room temperature. Although the researchers did not conduct further research in gas sensing field, the novel synthesis method, room temperature working temperature, and better selectivity all indicated that the synthesized materials should be further studied and explored.

**Figure 5:**
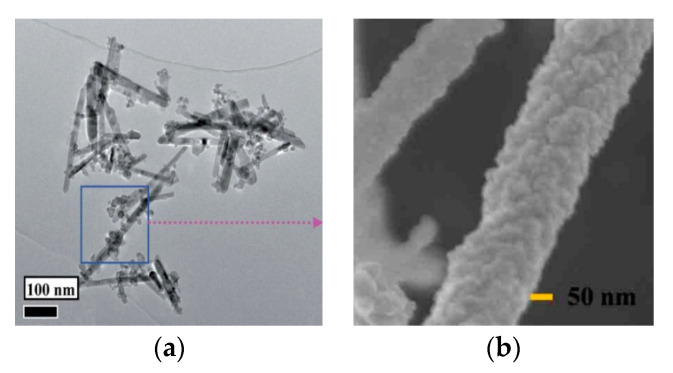
(**a**) A low-magnification TEM image of the as-prepared ZnFe_2_O_4_/ZnO nano heterostructures; (**b**) SnO_2_/ZnO nanowire [22,24].

The guide agent, a key element of the soft template method, is very widespread in the synthesis process of spinel morphology. Wang et al. [22] used FeSO_4_·7H_2_O and Zn(NO_3_)_2_·H_2_O as reaction raw materials, with cetyltrimethylammonium bromide (CTAB) as the directing agent. After usual steps, the final morphology of the ZnFe_2_O_4_/ZnO, which is shown in Figure 5a presented with a diameter of 9.4–34.7 nm and a length of 140–410 nm porous rod-like structures. The plausible mechanism of the rod-like structures is explained as the assistant of cation CTAB surfactants that be further explained as follows: under water bath conditions CTAB can help Zn(OH)_4_^2−^ combine with each other and decompose into ZnO nuclei which can self-assemble and grow into a rod-shaped structure in a specific axis orientation. Due to the unique structure formed by the surfactant and the role of the heterojunction, the ZnFe_2_O_4_/ZnO nano heterostructure had excellent gas sensitivities. Specifically, compared with the zinc oxide nanowire synthesized by Zou et al. [23], the heterojunction nanowire structure synthesized in this experiment can reach a gas response of 26.5 at a lower working temperature and a reaction time of 12 s, while the corresponding zinc oxide can only achieve a response of 9.1 to n-butanol at 320 °C. Wang et al. [24] had also done experiment using organic structure-directing agents to form rod-like structures. During the experiment, the researchers used SDSN as a directing agent, so that the material eventually formed a SnO_2_/ZnO nanowire structure shown in Figure 5b.

### 3.3. Nanosheets

The regular nanosheet structure can provide several active sites, expose effective crystal faces, and provide more electron transfer channels. All the tunes of the properties will effectively improve the gas performance of the material, attracting more people to study nanosheet morphology [25].

Hard template method, another broad application method, needs to use the material with fixed structure as a template to participate in the synthesis of the morphology and finally removed by some specific method. Wang et al. [26] chose KIT-6 as the template to synthesize porous ZnFe_2_O_4_ nanosheet structures and removed the hard template by 2 mol/L NaOH solution, leaving the ideal porous structure of ZnFe_2_O_4_. In experiments, it was found that the synthesized porous structure had good selectivity to acetone. Therefore, the authors speculated that the special mesoporous structure was an important factor in the selectivity to acetone.

**Figure 6:**
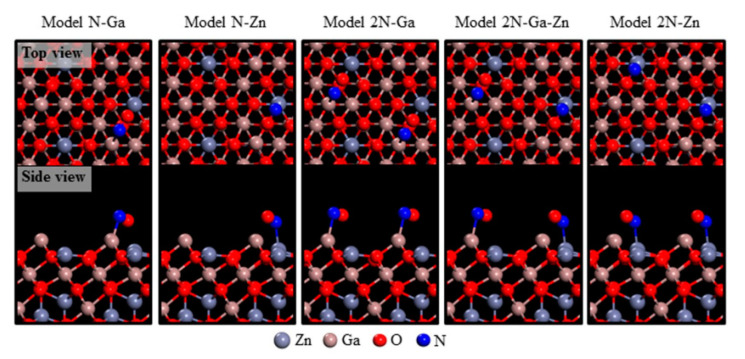
Atomistic representations of detailed N-Ga and/or N-Zn bonding arrangements pertaining to NO exposure on Ga-Zn-O-terminated ZGO (111) models. The atoms are represented by spheres: Zn (gray), Ga (brown), O (red), and N (blue) [27].

Wu et al. [27] synthesized ZnGa_2_O_4_ nanosheets which were able to detect NO, by using metal organic vapor deposition (MOCVD). Through SEM observation, it could be found that a layer of nanosheets was formed by the arrangement of spindle-shaped particles. The following gas sensitivity test showed that the material produced a response of 22.21 to 6.25 ppm nitric oxide gas at 300 °C, while compared with other gases like SO_2_ (125 ppm, 1.27) and CO (125 ppm, 1.06), the selectivity to NO is excellent. In order to further study the gas sensing mechanism, the researchers used first-principles reactions of gases with different surface structures, and proposed possible reaction processes by establishing five models, including N-Ga, N-Zn, and N-Zn-Ga, in specific model simulations, as shown in Figure 6.

However, the nanosheet tends to act as a transitional form and assemble or modify into another morphology. In order to prove this point of view, there are many paper data can be listed. Xu et al. [28] synthesized spherical-like nanostructures assembled from Zn_2_SnO_4_ nanosheets using Zn(CH_3_COO)_2_·2H_2_O and SnCl_4_·5H_2_O at a pH of 11 with ethylenediamine as the solvent. The experiment used synthetic materials to achieve a detection limit of 1 ppb for H_2_S at a working temperature of 133 °C with good selectivity.

**Figure 7:**
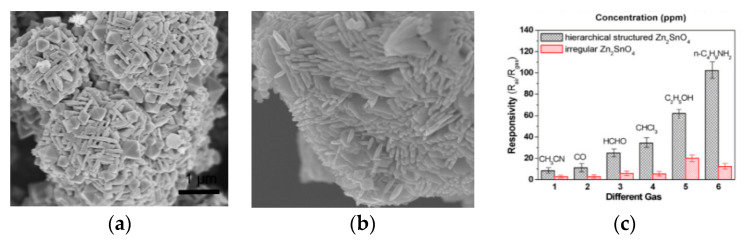
SEM images of (**a**) quasi-microspheres [22] (**b**) cube-like structures [29] (**c**) responsivity of Zn_2_SnO_4_-based sensors to different testing gases. The working temperature was kept at 350 °C (relative humidity: 50%) [29].

Jiang et al. [29] used raw materials and methods that are similar to the above-mentioned and synthesized a cubic nanostructure assembled from nanoplates without the calcination process. In the article, comparing the ordinary Zn_2_SnO_4_ with the assembled cubic structure, it can be found that the cubic structure significantly improved correspondingly through the response curve in Figure 7c. Although the specific surface area of the assembled cubic structure (26.99 m^2^/g) is smaller than that of the irregular Zn_2_SnO_4_ nanosheet structure (40.49 m^2^/g), the corresponding effect of the cubic structure is better. The result contrary to logic can be explained as the aggregation of gas molecules will not only affect the occurrence of high response, but also affect the response speed of the gas [30]. As a layered structure, the cube-like morphology has a clear porous structure, and the surface can have better accessibility than the sheet structure. At the same time, it can avoid particle consolidation and provide an effective path for the diffusion of the target gas. In addition, the obvious appearance of a (1 1 1) crystal plane may also provide active sites for gas adsorption, increasing the gas response of the overall material. More articles studied the combination of nanostructures forming into a bulk structure are listed in Table 1.

### 3.4. Nanospheres

The hollow sphere and the quasi-spherical structure increase the specific surface area and provide a smaller charge transfer distance [34] with a higher permeability [35] because of the unique morphology. Therefore, the hollow nanospheres are more suitable for the detection of various gases than the solid structure.

(10) $$2{\mathrm{NO}}_{2(\mathrm{gas})}+{{2\mathrm{OH}}^{-}}_{\left(\mathrm{abs}\right)}\to {\mathrm{NO}}_{2}{{}^{-}}_{\left(\mathrm{abs}\right)}{+\mathrm{NO}}_{3}{{}^{-}}_{\left(\mathrm{abs}\right)}{+H}_{2}O_{\left(g\right)}$$

**Figure 8:**
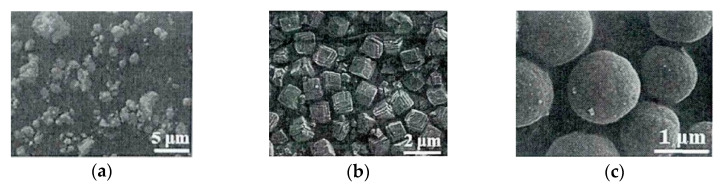
SEM images under different reaction conditions (**a**) Molar ratio of ethanol to EG = 1:1 and 180 °C for 4 h; (**b**) EG alone and 180 °C for 10 h; (**c**) Molar ratio of ethanol to EG = 1:1 and 180 °C for 10 h. [38].

Yang [36] synthesized the porous nanosphere structure of CuFe_2_O_4_ by solvothermal method. Comparing the pure CuO and α-Fe_2_O_3_ nanoparticle sensors, the response of the synthesized porous structure to toluene at 250 °C can reach at 20.1, which was nearly 5.4 times that of CuO and α-Fe_2_O_3_. This proves the feasibility of porous materials in gas detection. Zhou et al. [37] used ethanol and ethylene glycol (EG) as solvents to synthesize a ZnFe_2_O_4_ porous nanosphere structure composed of nanoparticles by a simple solvothermal method. After testing, the synthesized material was able to detect acetone at a minimum of 800 ppb, and the response could reach 1.5, refreshing the lower limit of acetone detection in the spinel material sensing field. In the article, Liu [38] synthesized ZnCo_2_O_4_ porous hollow spheres by using a series of methods of controlling variables to find the appropriate reaction conditions in the experiment. Through observing the following SEM image in Figure 8, it could be found that the material is gradually formed from irregular particles into a hollow sphere structure with a consistent shape. In addition, through many experiments, it is possible to discover that the mixed solution of ethanol and EG is often used as a solvothermal reaction environment.

Zhang et al. [39] synthesized hollow NiFe_2_O_4_ octahedral structure using self-sacrificial template method with dimethylformamide (DMF) and ethanol as solvents with terephthalic acid (PTA) and diaminobenzidine (DAB) as guiding agents. In order to prove the actual effect of the two guiding agents, the authors experimented by adding PTA and DAB separately. The results showed that only sheet-like and regular structures were synthesized, still it could not further form into an octahedral hollow structure, presenting both directing agents as necessary for the synthesis of morphology.

### 3.5. Nanoflowers

The 3D flower-like structure provides an inward diffusion path for the gas, so it is capable of improving the sensing performance effectively. Chen et al. [31] demonstrated the enormous gap between the Zn_2_SnO_4_ nanoflower structure and the solid structure of the sphere. At an operating temperature of 380 °C, the response of the nanoflower structure to 50 ppm ethanol could reach 30.4, which was three times the corresponding structure.

**Figure 9:**
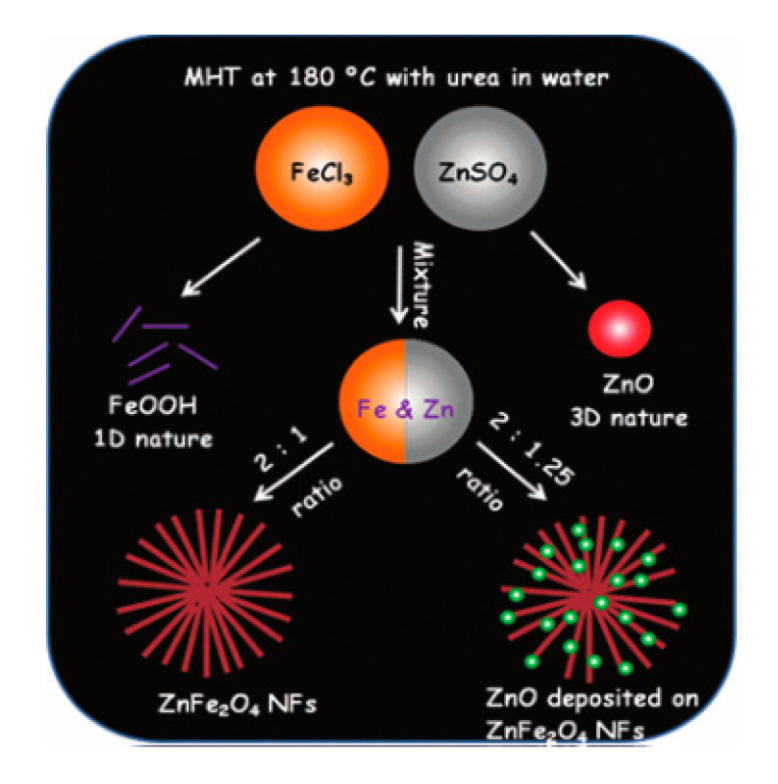
Pictorial presentation of the synthetic strategy of the as-synthesized materials at different reaction conditions [40].

Sahoo et al. [40] used the modified hydrothermal (MHT) to achieve the synthesis of two type flower-like structures by choosing different synthesis strategies. The MHT under the action of urea made the reaction process reactant Fe(OH)_3_ dehydrogenated into (FeOOH). After adjusting the molar ratio of Zn to Fe, two different forms of nanoflowers were finally generated, and detailed processes are clearly shown in Figure 9. In addition, Sahoo also mentioned the application of ZnFe_2_O_4_ nanoflowers in other fields like mimicing peroxidase activity in this paper. This experimental result showed that ZnFe_2_O_4_ was helpful for the selective detection of H_2_O_2_ and Hg^2+^ ions in the solution by the naked eye as well.

Liu et al. [41] synthesized ZnFe_2_O_4_/ ZnO nanoflowers by mild hydrothermal method. The first was to synthesize the ZnO nanoflowers at 80 °C, then rinsed the nanoflowers three times with the configured Fe(NO_3_)_3_ solution, and finally burned the sample at 500 °C to form ZnFe_2_O_4_/ZnO nanoflowers. In the gas property test, the sample synthesized this time produced an 8.3 response to 50 ppm acetone at an operating temperature of 250 °C; in comparison, pure zinc oxide had only a 5.2 response to 100 ppm acetone at 300 °C. The results showed that the composite nanoflower structure had a good improvement in working temperature and gas response.

### 3.6. Core-Shell Structures

Core-shell structure, a morphology proposed later, makes the spinel-type sensitive materials widely applied in the fields including supercapacitor electrodes [42,43], ions batteries [44], catalysts [45], and sensing [1]. Since most of the core-shell structure is derived from the nanosphere structure, it can be regarded as an impact on morphology, which will be partly introduced in the enhancing chapter.

Hu et al. [46] used the solvothermal self-assembly method for the first time to synthesize litchi-shaped ZnO/ZnFe_2_O_4_ core-shell hollow microspheres. Via the SEM images, it could be found that the ZnO/ZnFe_2_O_4_ product displayed as the required core-shell structure, and the response to 100 ppm acetone reached 33.6 at 280 °C, which was nearly five times that of the ZnO (6.0) and twice that of the pure ZnFe_2_O_4_ (17.3).

**Figure 10:**
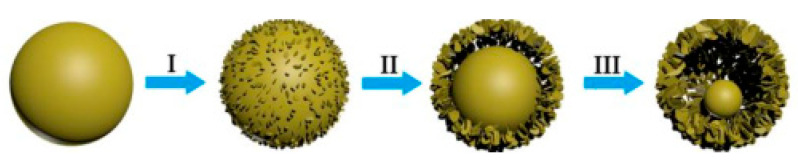
Ostwald ripening process of core-shell ZnFe_2_O_4_ [47].

**Figure 11:**
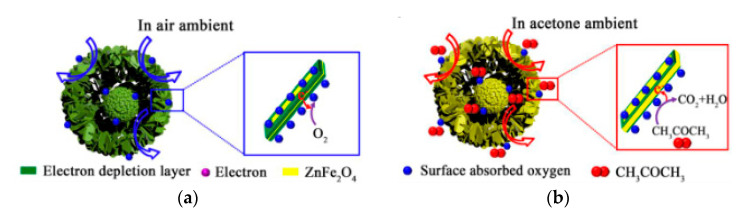
Schematic diagram of possible reasons why ZnFe_2_O_4_ core-shell microspheres exhibit higher acetone response (**a**) contact with air; (**b**) contact with the aimed gas [47].

Zhou [47] synthesized the core-shell ZnFe_2_O_4_ by increasing the reaction time from 1 h to 21 h in the experiment and observed the morphological changes which are shown in Figure 10. From the figure, as the increase of reaction time went, due to the occurrence of an Ostwald ripening process [48,49,50], the product gradually changed from a solid ball to a core-shell structure, and reached the ideal porous core-shell structure at 21 h. The author made a guess about the possible working process, which is shown in Figure 11.

Compared with the structure of zinc ferrite mentioned in other similar papers, the synthesized core-shell structure exhibited good gas sensitivities. This material produced a response of up to 28.3 to 50 ppm of acetone at an operating temperature of 200 °C, while the pure zinc ferrite nanocube [51] only produced the response of 18.5 to 1000 ppm of target gas and the graphene-zinc ferrite composite structure [52] produced a response of 9.1 to 1000 ppm acetone, respectively. The obvious difference in performance showed that the material had a strong detection ability for acetone, even far exceeding the composite structure.

## 4. Enhancing

In the past research process, the direction of improvement on spinel materials (especially ZnFe_2_O_4_ [19]) can be mainly divided into two categories: the improvement of the morphology itself and the doping with other materials. Because doping is an important and effective route to promote the properties of semiconductors [53,54,55,56], more researches are conducted. Compared with single metal oxidation, spinel materials are able to be modified by some common metals. Moreover, this modification can effectively replace the original metal ions and largely change the morphology and performance of the original material [57]. Several improving methods are stated as follows.

### 4.1. Reaction Process

**Figure 12:**
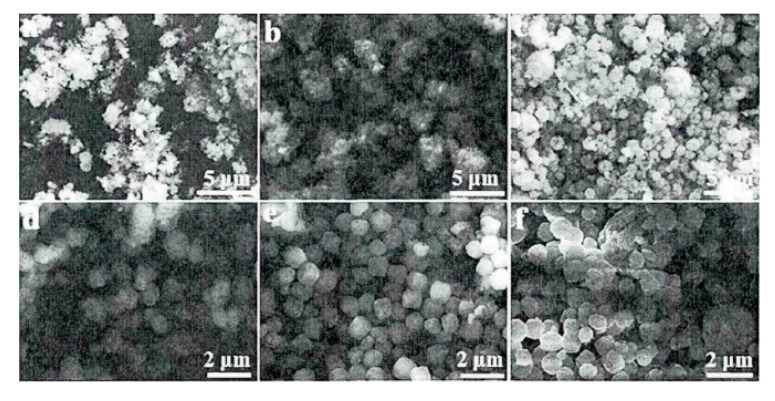
SEM picture of the effect of reaction time on morphology (**a**) 0 h; (**b**) 2 h; (**c**) 4 h; (**d**) 6 h; (**e**) 8 h; (**f**) 15 h [58].

Various reaction factors in the reaction process have a key influence on the formation of the material structure, such as the molar ratio of the reactants, the reaction time, pH value, and grinding, all of which will confirm the gas-sensitive potential of the morphology. In the following, this point will be proven through several sets of experiments. In the process of synthesizing ZnFe_2_O_4_ nanosphere by hydrothermal method, Liu [58] analyzed the influence of the solvent system, reaction temperature and reaction time on morphology through multiple sets of experiments. Typically, the author set the reaction time to four stages of 0, 4, 8, and 15 h to obtain the effect of time on morphology by analyzing the SEM image in Figure 12. The results showed that too short a reaction time will not form a regular morphology while too long a reaction time will destroy the morphology and cause the nanoparticles to reunite.

**Figure 13:**
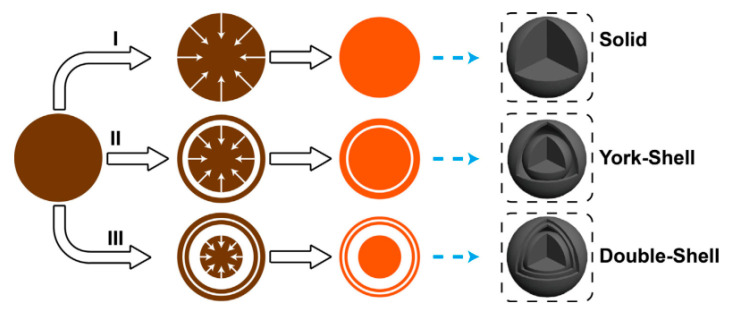
Formation process of ZnFe_2_O_4_ double-shell, York-shell and solid microspheres. I heating rate: 1 °C/min; II heating rate:20 °C/min; III directly placed in 350 °C [59].

**Figure 14:**
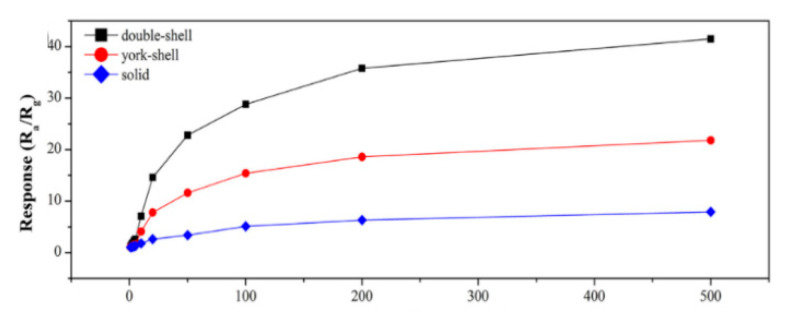
Response of ZnFe_2_O_4_ double-shell, core-shell and solid microspheres sensor as a function of acetone concentration [59].

Qu et al. [59] synthesized ZnFe_2_O_4_ with double shell structure using 0.25 mmol zinc nitrate and 0.5 mmol ferric nitrate as raw materials, and a mixed solvent of glycerol and isopropanol as reaction environment. In the annealing process, three products with different morphologies were formed by controlling various heating rate upon 1 °C/min, 20 °C/min to directly 350 °C. From Figure 13, it can be found that as the heating rate is gradually accelerated, the morphology gradually changed from a solid ball to a double-shell structure. Observing the response of the three structures to acetone in Figure 14, it was seen that the increase in the number of shells had a good improvement in the response property of the gas. The impact on sensing properties are due to the porous surface of the double-shell structure, which can provide more active sites, adsorbing more oxygen without preventing gas diffusion.

**Figure 15:**
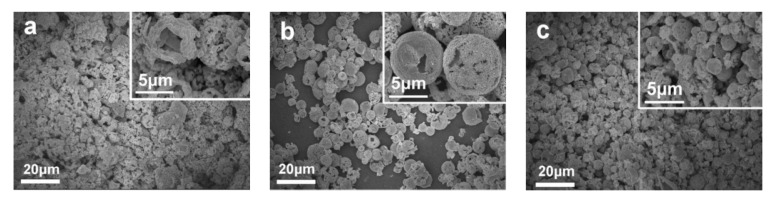
The SEM patterns of NiMn_2_O_4_ with different dripping rate. (**a**) NiMn_2_O_4_ (1 s), (**b**) NiMn_2_O_4_ (2 s) and (**c**) NiMn_2_O_4_ (3 s) [60].

Guan et al. [60] studied the correlation between the dripping speed and morphology of the material. Three control groups were set up throughout the experiment, and the liquid drip rate was set to 1 s/drop, 2 s/drop, and 3 s/drop respectively. By observing the SEM pattern difference of the three groups of materials in Figure 15, it can be found that the morphology in the 3 s/drop is the most complete and most suitable for gas detection. Guan proposed a probable reason for this phenomenon. When the dripping speed is too fast, the evaporation speed of the water cannot keep up with the dripping speed, which will cause the hollow ball to break. When the dripping speed becomes slower, the water can be dispersed into smaller droplets that support a transformation to a complete and smaller spherical structure.

It is worth noting that grinding also influences morphology. In the experiment, Yang [61] conducted a grinding operation on the synthetic product. From the comparison of the SEM images before and after, it can be found that the grinding destroyed the original spherical structure, and the material has agglomerated. Therefore, in the experiment, the grinding operation should be carried out selectively to keep the most rational appearance.

### 4.2. Graphene

Graphene oxide, a common material used to improve gas sensing property, has an obvious enhancing effect. As a p-type semiconductor, the two-dimensional GO material can effectively reduce the working temperature of the gas sensor and use the oxygen-containing functional groups on the surface to adsorb the target gas molecules [62,63].

**Figure 16:**
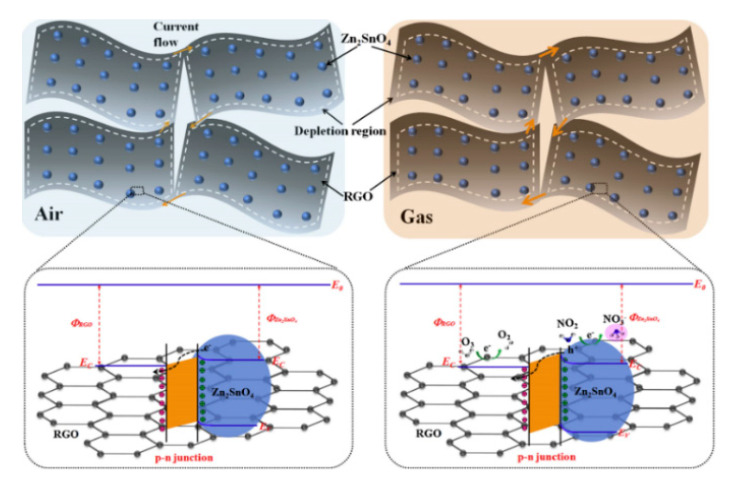
The representative models of Zn_2_SnO_4_-RGO hybrids: schematic illustration of the energy band structures and the form of p-n heterojunction in air and oxidizing gases [64].

Wang et al. [64] used Hummers’ method [65] with the hydrothermal method to obtain the structure of Zn_2_SnO_4_ particles attached to RGO nanosheets and tested the NO_2_ in a humid environment. The common NO_2_ sensor is greatly affected by humidity and the specific performance is that the higher the humidity, the lower the response [66,67,68,69], while the synthesized material could achieve a response of 5.97 to 1 ppm NO_2_ at the operating temperature of 30 °C under an environment of 80% rh. Laboratory finding concluded into the higher the humidity, the better the performance of this sensor, and this is mainly because the adsorbed water makes a great effort in the chemical adsorption process of the target gas molecule [70] which promotes the sensing reaction. Further, the participation of graphene reduces the operating temperature of the spinel sensor, enabling the sensor to perform gas detection at room temperature; the porous structure of graphene provides more sites for the gas to adsorb and increases gas response [71]. In addition, the formation of a p-n heterojunction can also increase the gas sensing performance, and the principle shown in Figure 16 will soon be explained in the heterojunction part.

**Figure 17:**
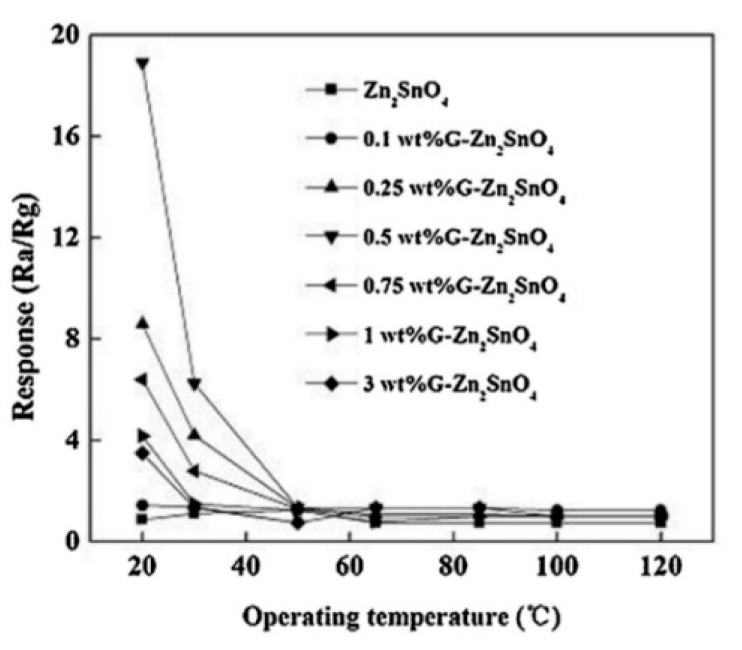
The responses to 1000 ppm formaldehyde vapor of graphene-Zn_2_SnO_4_ with different graphene content (0.0, 0.1, 0.25, 0.5, 0.75, 1, and 3 wt%) prepared at 200 °C [72].

Chu et al. [72] studied the influence of graphene content on the gas-sensing properties of zinc stannate. By changing the proportion of graphene, it could be found from the response curve of Figure 17 that each material had the best response at room temperature (20 °C). Compared with the higher operating temperature (200 °C or higher) of other undoped zinc stannate materials [31,73,74,75], the result showed that graphene has a good improvement effect on working temperature. At the same time, it can be seen intuitively in the curve that the best response (18.9) to formaldehyde gas was when the graphene doping content is 0.5 wt%. Chu proposed a reasonable explanation for this phenomenon in that the addition of graphene created a p-n heterojunction at the interface of the materials, which effectively improved the gas sensing characteristics, but when too much graphene was added to the material, a “shortcut” would be formed between the graphenes, which weakened the affection of the heterojunction.

### 4.3. Heterojunction

Heterojunction refers to the interface area formed by the contact of two different semiconductors [1]. Due to the different work functions of the two semiconductors, in order to enable the Fermi level to be at the same level, electrons will undergo corresponding electron transfer between the two which depends on the material’s type. Meanwhile, electron depletion, formed at the junction of the two layers, will help to increase the gas sensing properties. According to the type of two semiconductor materials, heterojunctions are divided into three categories: n-n-type heterojunctions, p-p-type heterojunctions, and p-n-type heterojunctions. Part of the literature results are summarized in Table 2.

**Figure 18:**
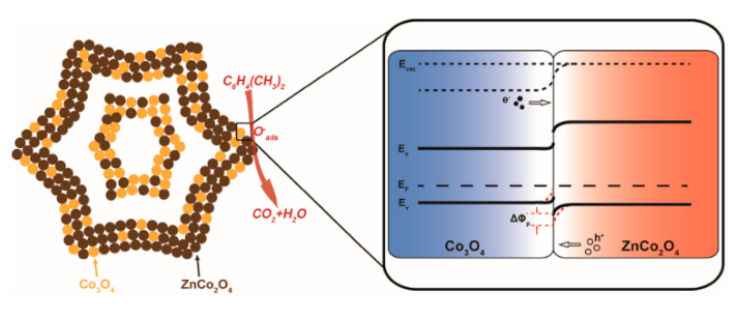
Schematic illustration of the proposed gas sensing mechanism of Co_3_O_4_/ZnCo_2_O_4_ composite hollow nanostructure [82].

Qu et al. [82] considered that Co_3_O_4_ and ZnCo_2_O_4_ have the same crystal structure, so a reasonable material combination is possible to be made. Qu used molecular sieve imidazole framework-67 (ZIF-67) as the self-sacrificial template and the Co_3_O_4_/ZnCo_2_O_4_ composite hollow nanostructure was obtained through transformation and heat treatment. The composite material conjectured composite structure shown in Figure 18 presented a peculiar rhombic dodecahedron hollow structure, which provided an effective means for gas diffusion. In addition, the p-p type heterojunction formed in the material with the thin shell close to the Debye-length outside both helped to improve the overall gas sensitivity, so that the synthesized material produced a 16.3 response to 100 ppm at a working temperature of 255 °C.

### 4.4. Metal Doping

Noble metal doping is a conventional method which effectively improve the gas-sensing properties of semiconductors and has been effectively applied to single oxide materials with multiple morphologies [83]. Due to the particularity of spinel-type materials, it can be doped with common metals also, which will undergo substitution between elements [84], and modify the physical properties of the morphology to achieve the purpose of improving performance. Next, the metal doping enhancement will be described from two aspects.

#### 4.4.1. Noble Metal

As a common correction method, doping noble metals has been studied for a long time. The noble metals mainly refer to gold, silver, and platinum group metals (including Ru, Pt, Pd, etc.) which have gorgeous lusters and are quite resistant to most of the chemicals under normal circumstances [1].

**Figure 19:**
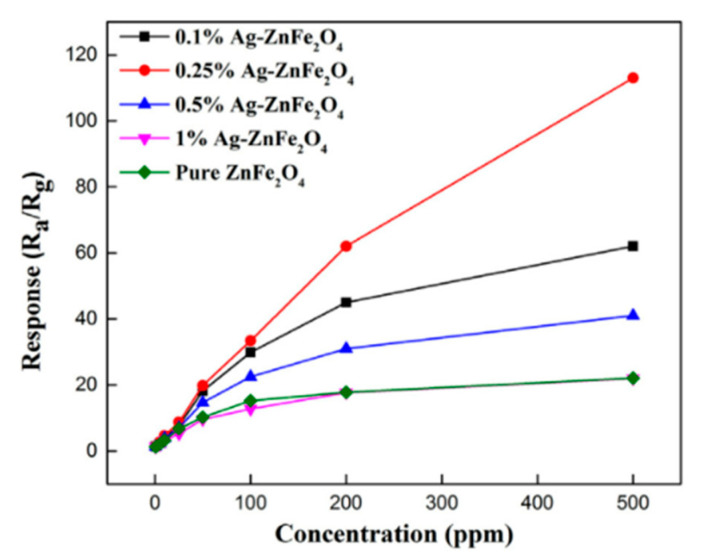
The responses of Ag-ZnFe_2_O_4_ sensors to 0.8–500 ppm acetone vapor [83].

Zhang et al. [83] used Zn(CH_3_COO)_2_·2H_2_O and Fe(NO_3_)_3_·9H_2_O as raw materials to synthesize a hollow spherical structure formed by stacking zinc ferrite nanosheets, and set 4 groups of different Ag contents (1, 0.5, 0.25 and 0.1 wt%) experiments to study the influence trend of different content of precious metals on the gas sensitivity of materials. By observing the test curves in Figure 19, the performance was best when the doping amount is 0.25 wt%. At this time, the response/recovery time (17 s/148 s) of the material modified with silver was compared with that of the unmodified material (34 s/307 s) had a significant improvement. In studying the influence of different silver content on the gas-sensitivity performance, Zhang found that the response continued to increase with the increase of the content, while there was a significant drop after reaching 0.25 wt%. Zhang made a reasonable explanation for this phenomenon suggesting that excessive additives (silver nitrate) destroyed the flaky structure formed by ZnFe_2_O_4_, reduced the active sites on the surface, and caused a corresponding decrease. Therefore, when performing the noble metal doping, it is necessary to consider the influence of doping amount on gas sensitivity.

Many articles [53,84,85,86,87] have studied the sensitization of precious metals, and it is currently believed that the performance can be affected by the following two aspects.

**Figure 20:**
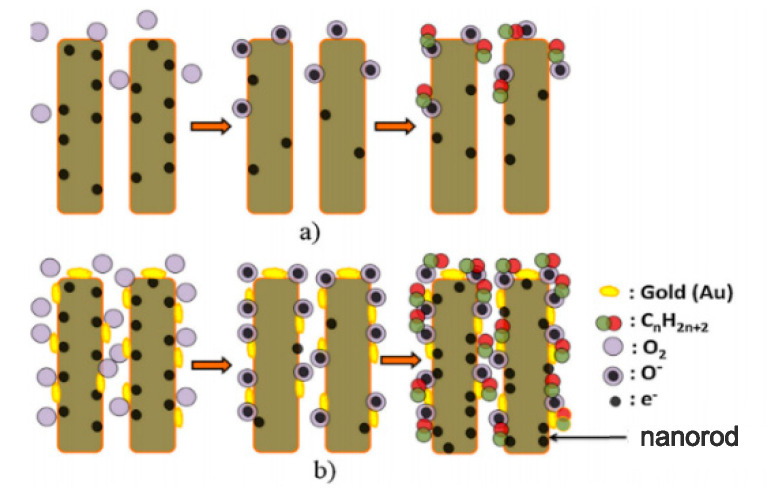
Schematic diagram of gas sensing (**a**) unsensitized nanorods (**b**) Au-sensitized nanorods [53].

**Figure 21:**
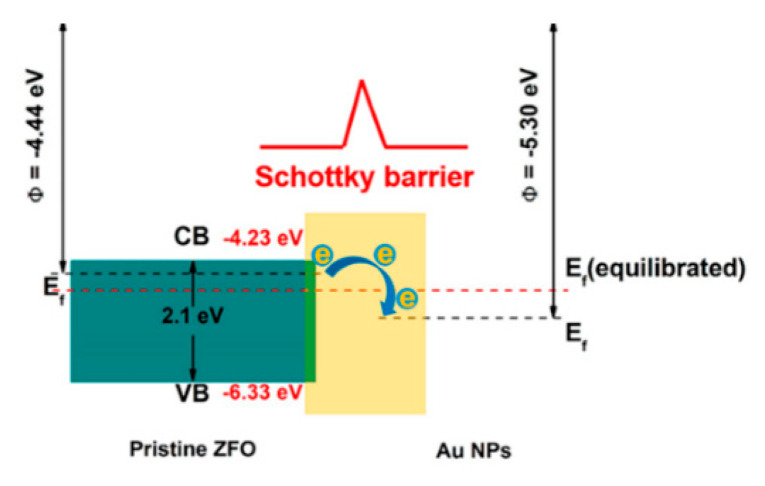
Sensing mechanism of the Au-ZnFe_2_O_4_ core-shell-sphere-based sensor in energy band diagram [90].

(1) Chemical sensitization: Precious metals have the characteristics of adsorbing oxygen molecules and target gases in the surrounding area. Under the same circumstances, precious metal-modified semiconductor devices tend to provide more active sites, forming more adsorbed oxygen which affects the initial resistance; at a certain temperature, it is worth noting that some metals have a special ability on attracting the specified gas, increasing the number of aimed gas adsorbed on the metal surface first, diffusing to the outside of the semiconductor later [88,89]. The sensitization process can be understood simply through Figure 20.

(2) Electronic sensitization: Schottky junction will be formed on the contact surface of metal nanoparticles and semiconductor material. Take Au and ZnFe_2_O_4_ core-shell structure as an example for the specific explanation [90]. The work functions of ZnFe_2_O_4_ and Au are 4.4 eV and 5.3 eV, respectively, which means that the Fermi energy level of Au is higher. In order to achieve a unified Fermi energy level, as shown in Figure 21, part of the electrons will be transferred from the semiconductor to the noble metal, so that the combined material has a higher initial resistance and is more sensitive to gases. In terms of data, the sensitization effect was shown as the original ZFO produced a 20.2 response to 30 ppm chlorobenzene (CB) gas, while the gold-modified ZFO produced a 90.9 response to this concentration of gas.

Since the Schottky junction and the heterojunction are both effective methods for improving the property, some people jointly applied the two to form nanostructures for gas testing. Li et al. [91] synthesized a hollow mesh structure of Au-doped ZnO/ZnFe_2_O_4_ and tested it on acetone. The material synthesized this time is five times the response of pure ZnO, showed the potential of the nano-meshes on acetone detection and provided new thinking that can be referenced for high-performance acetone gas sensors.

#### 4.4.2. Metal Element Replacement

Inside the spinel structure, there are numerous voids in the tetrahedron and octahedron formed by close packing of oxygen. When the voids are doped by other elements or the original elements are replaced, it will have a great impact on the gas sensitivity of the material [92,93,94]. At present, people mainly study the doping or modification between non-noble metals and the spinel structure. Further, many studies have shown that the addition of non-metals can effectively change the structure of the material, thereby achieving the purpose of affecting gas sensitivity.

**Figure 22:**
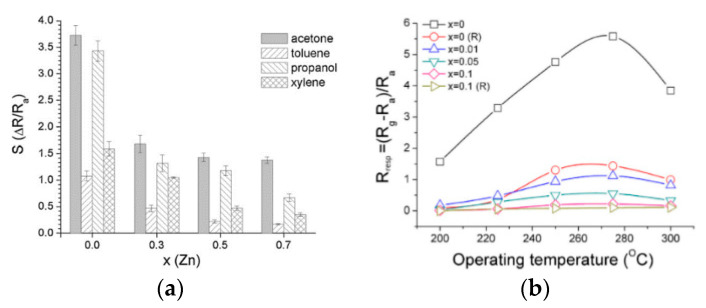
(**a**) Response of Ni_1−x_Zn_x_Fe_2_O_4_ compositions for various aimed gases [99] (**b**) Response of Ni_1−x_Co_x_Fe_2_O_4_ compositions for 500 ppm acetone in various operating temperature [100].

Deraz et al. [95] used the combustion method with changing the content of added zinc nitrate and finally synthesized the Zn/CoFe_2_O_4_ porous structure. By observing the analysis of the XRD diagram, it can be found that element replacement had occurred. Based on experience, researchers used the diffraction intensity of the (3 1 1) plane as a measure of crystallinity. With the increase of zinc content, the height of XRD main peak continued to rise, reaching the original 135%, which phenomenon was positively caused by the substitution of Zn for Co. At the same time, the increase in other data indicators such as the crystallite size (d), lattice constant (a) and unit cell volume (V) also illustrated the redistribution of cations between octahedral and tetrahedral sites. The replacement of zinc ions produced ZnFe_2_O_4_, which inhibited the continued growth of nuclei, made the particle size smaller and improved the gas-sensitive response by increasing the specific surface area conductively. Many articles on metal doping have proven that proper doping can reduce particle size and improve gas sensing performance [96,97,98]. A few experiments [99,100] have proved that when the selected doped metal is inconsistent with the original semiconductor material, there will be a reduction in sensitivity such as the limitation of the sensor’s conduction function, making the gas-sensing properties of the composite material inferior to those when it is not doped. The specific impact can be seen in Figure 22.

## 5. Conclusions

This article mainly introduces four aspects, including the basic introduction of the spinel structure, the gas sensing mechanism, six common morphologies including nanoparticles, nanosheets, core-shell structures, etc., and four methods to improve performance.

A consistent nanostructure and morphology are vital for gas sensing performance, so spinels with a controllable morphology are gradually studied. In the synthesis, the hard template method and the soft template method are mainly used because both methods can play a decisive role in morphology. When the direct synthesis cannot continue to improve the gas-sensitive performance, doped material can be considered.

At present, only some of the common spinel structures have been studied to a certain extent. More spinel structures are rarely used in gas sensing because of high synthesis temperature and low response. Therefore, the current ability to control the morphology of the material effectively and the improvement of the gas response is the direction that should continue to be strengthened for spinel-type materials. In addition, the spinel structure is relatively complex, and there is no perfectly accurate theory for the gas sensing mechanism, so in-depth research should be carried out on the theoretical model.

## Figures and Tables

**Table 1:** Different research on assembling structures towards various aim gas

Hierarchical Sensing Material	Building Block	Temp.	Conc.	Response	Refs.
hierarchical Zn_2_SnO_4_ quasi-microspheres	nanosheet	133 °C	H_2_S 100 ppm	144.3	[28]
hierarchical Zn_2_SnO_4_ cube-like structures	nanoplate	350 °C	n-butylamine 400 ppm	102.2	[29]
hierarchical Zn_2_SnO_4_ 3D flower-like structures	nanorod	380 °C	ethanol 50 ppm	30.4	[31]
Zn_2_SnO_4_ hollow microcubes	nanotube	260 °C	acetone 200 ppm	141.7	[32]
Zn_2_SnO_4_ octahedral structures	nanosheet	250 °C	ethanol 100 ppm	87.3	[33]

**Table 2:** Heterojunction structures.

Type	Sensing Material	Aiming Gas	Conc.	Temp.	Response	Ref.
n-n	ZnFe_2_O_4_/ZnO nanoparticle decorated rod-like structure	N-butyl alcohol	200 ppm	260 °C	26.5	[22]
	ZnSnO_3_/Zn_2_SnO_4_ flower-like structure	phenylamine	20 ppm	260 °C	12.1	[76]
p-n	ZnO/ZnFe_2_O_4_ actinomorphic flower-like structure	NO_2_	10 ppm	200 °C	250	[77]
	ZnO/ZnCo_2_O_4_ hollow core-shell nanocages	xylene	100 ppm	320 °C	34.26	[78]
	PPy/Zn_2_SnO_4_ nanocomposite	NH_3_	100 ppm	room temp.	82.1	[79]
	ZnFe_2_O_4_/SrTiO_3_	--	--	--	--	[80]
p-p	CuO/CuCo_2_O_4_ nanotubes	n-propanol	10 ppm	room temp.	14	[81]

“--” in the table means that the performance is not mentioned in the text.
